# HLA-A29 and Birdshot Uveitis: Further Down the Rabbit Hole

**DOI:** 10.3389/fimmu.2020.599558

**Published:** 2020-11-11

**Authors:** Jonas J. W. Kuiper, Wouter J. Venema

**Affiliations:** ^1^Department of Ophthalmology, University Medical Center Utrecht, University of Utrecht, Utrecht, Netherlands; ^2^Center for Translational Immunology, University Medical Center Utrecht, University of Utrecht, Utrecht, Netherlands

**Keywords:** birdshot, HLA-A29, antigen presentation, uveitis, peptide

## Abstract

HLA class I alleles constitute established risk factors for non-infectious uveitis and preemptive genotyping of HLA class I alleles is standard practice in the diagnostic work-up. The HLA-A29 serotype is indispensable to Birdshot Uveitis (BU) and renders this enigmatic eye condition a unique model to better understand how the antigen processing and presentation machinery contributes to non-infectious uveitis or chronic inflammatory conditions in general. This review will discuss salient points regarding the protein structure of HLA-A29 and how key amino acid positions impact the peptide binding preference and interaction with T cells. We discuss to what extent the risk genes *ERAP1* and *ERAP2* uniquely affect HLA-A29 and how the discovery of a HLA-A29-specific submotif may impact autoantigen discovery. We further provide a compelling argument to solve the long-standing question why BU only affects HLA-A29-positive individuals from Western-European ancestry by exploiting data from the 1000 Genomes Project. We combine novel insights from structural and immunopeptidomic studies and discuss the functional implications of genetic associations across the HLA class I antigen presentation pathway to refine the etiological basis of Birdshot Uveitis.

## Introduction Into Birdshot Uveitis

Birdshot Uveitis (also known as *Birdshot chorioretinopathy* or *Birdshot retinochoroidopathy*) is a well-characterized form of autoimmune uveitis (inflammation of the uveal layer of the eye) mostly known for its ovoid light lesions, which appear ‘shotgun pattern’-like distributed along the vascular arcades in the back of the eye (i.e., the ‘fundus’ of the eye where these lesions are visible by photography) (1). Inflammation and extensive depigmentation of the choroid, macular edema, peripheral ischemia (2), degeneration of the retina, and the progressive formation of thin layer of scar tissue on the retina (“epiretinal membrane”) (3, 4), progressively impair vision in a substantial proportion of patients. BU is unusual in the young (5) and typically affects patients over 50 years of age of Western-European ancestry, with more women than men affected (6). Long-term systemic corticosteroid-sparing immunomodulatory therapy is the mainstay of treatment (7, 8), but a fraction of patients may exhibit a more benign disease course that does not require systemic therapy (9). Histopathology studies of eye tissues and modern imaging technologies show that early lesions are located deep inside the vascular layer of the eye (the “choroid”) between the retina and the white outer layer of the eyeball (sclera). In the choroid, the large-vessel layer (choroidal stroma)—densely populated with pigmented “melanocyte” cells—shows abnormalities before the characteristic fundus lesions are visible (10, 11). Because BU shows early inflammation of the choroidal stroma (12), Herbort and associates proposed to classify BU as a primary *stromal choroiditis*, together with *Vogt-Koyanagi-Harada* (VKH) disease. VKH is a condition characterized by chronic inflammation toward melanocytes that affect multiple parts of the body, including the choroidal stroma and the larger choroidal vessels (11, 13). In VKH no retinal involvement at early stages of disease are noticeable. In contrast, retinal inflammation (e.g., leakage of vessels) is an early clinical characteristic of BU (14), which suggests that retina involvement is not merely the result of inflammation spilling over from the choroid. However, the cause and interdependence of the retinal and choroidal inflammation are unknown, which is reflected in the use of multiple terms to define the eye condition; birdshot retinitis, birdshot *chorioretinopathy*, or birdshot *retinochoroidopathy*. For lack of understanding the disease pathology, here the broader term “birdshot uveitis” was chosen.

Microscopic anatomy studies (or *histological* studies) of eye tissue of patients with BU are scarce because of the rarity of the condition (estimated 1 to 5 cases per 500,000) (6, 15). The most recent histological study by Sohn and coworkers (16) in a patient with end-stage BU showed extensive degeneration of the retina and near complete loss of choroidal layers and the retinal pigment epithelium, a highly specialized cell layer critical to the homeostasis photoreceptors of the retina. Changes in retinal pigment epithelium are also evident by retinal imaging in patients with established disease (17). Each of the histological studies show massive infiltration of blood leukocytes into the choroid and retina layers; mostly T lymphocytes not only express the glycoproteins CD4 (“T helper” cells) and CD8 (“Cytotoxic” T cells) (16, 18, 19) but also relatively increased numbers of other immune cells, such as myeloid cells and B lymphocytes. The cases in two of these studies were remarkable for a history of malignant melanoma, but evidence that directly links melanoma to BU is lacking. At most, the evidence is circumstantial, such as “Birdshot-like disease” in melanoma cancer patients that develop autoimmune uveitis due to checkpoint inhibitor therapy (a treatment setting T cells free to kill tumor, but also normal tissue) (20) or the presence of blood antibodies that can bind to proteins in melanoma tumor cell lines (21). These phenomena may be explained by the fact the proteins involved in immunity toward melanoma are also expressed in normal melanocytes (22) and may actually support that choroidal melanocytes are among target cells deliberately attacked by the derailed immune system in BU.

## The Genetic Association With HLA-A29

Short after BU was first described in 1980 (23), the unusually strong genetic association of the Human-Leukocyte Antigen A*29 (HLA-A*29) with BU was discovered in 1982 by Nussenblatt and coworkers (24). HLA-A29-positive testing is now widely considered critical to diagnosis and led key opinion leaders in the field propose to rename the condition to “HLA-A29 uveitis” (25). HLA-A*29 is one of the hundreds of variants of the *HLA-A* gene that together with different versions of *HLA-B* and *HLA-C* genes form the *HLA class I complex* of functionally related proteins in humans. The *HLA-A* gene encodes slightly different versions of a the cell-surface protein HLA-A. Like other HLA class I proteins, HLA-A plays a central role in the immune system by instructing immune cells (e.g., cytotoxic T cells) if a cell must be destroyed because it is infected by foreign invaders (e.g., a virus) or when a cell has become cancerous after mutation of the DNA (26). In most cells of the body, HLA-A achieves this by constant sampling of protein fragments from foreign invaders or self-proteins (termed “antigenic” peptides, or antigens in short) from the inside of the cell and “presenting” these peptides on the outside of the cell for scrutiny by surveilling immune cells (26, 27). This “antigen presenting pathway” is critical to monitor cellular integrity and is based on differentiating “self” from “non-self” (pathogen) or “altered-self” (cancer) (26). Aberrant function of this pathway can result in persistent infection, cancer or autoimmune disease (27).

Because all patients with BU carry a copy of the HLA-A29 *allele* (the term for “gene variant”), it is considered to be critically involved in the unidentified disease mechanisms (1). This is supported by rare familiar cases of BU that show that all cases with the eye phenotype are also HLA-A29-positive (28). Also, the allele frequency of HLA-A29 is high in Western-European countries (29), where also the vast majority of BU patients are reported in Europe, while BU is anecdotally reported in populations with low occurrence of HLA-A29 (30, 31). How exactly HLA-A29 contributes to eye inflammation is unknown, but several unique properties of HLA-A29 distinguish this allele from others *HLA-A* alleles in the population; A gel electrophoresis study from 1992 indicated that HLA-A29 in cases is identical to unaffected controls that carry HLA-A29 (~5–10% of the Western-European populations) (32), which is supported by small DNA sequencing studies (29). In two genome-wide association studies (33, 34), we used detailed genetic analysis of HLA alleles in BU cases that revealed that the main risk allele for BU is HLA-A*29:02, the most common HLA-A29 allele in Europe. These studies further ascertained that other associations in the *MHC* locus (the DNA region where HLA genes are embedded) are a result of positive linkage disequilibrium (LD) with HLA-A29. In other words, near-by gene variants such as for example the HLA-B*44 allele are often (yet not always) inherited together with HLA-A29 but most likely not relevant for the disease. One study of a murine model in which a copy of HLA-A29 DNA from a BU patient was genetically expressed initially showed an eye disease similar to BU (35), but in a later underappreciated study, the mice strain used for the BU model was found to harbor a wide-spread and previously unnoticed genetic mutation that causes retinal degenerative disease (not uveitis) that also affected the control mice (36). This supports that the HLA-A29 allele itself is not sufficient and that the susceptibility to BU is mediated by additional etiological triggers. This also fits the observation that HLA-A29 is a common allele (~10% of the Western-European population is HLA-A29-positive), but BU is a rare condition (~250 cases in the Netherlands at 17 Mill. citizens as of June 2020). Also, HLA-A*29:02 is also very common among specific ethnic groups of non-European ancestry where BU has not been reported, such as the South African Zulu (37) or the Luhya in Webuye of Kenya in Africa (~10% HLA-A*29:02-positive individuals) (38, 39).

## The HLA-A29 Protein Structure

**Figure 1:**
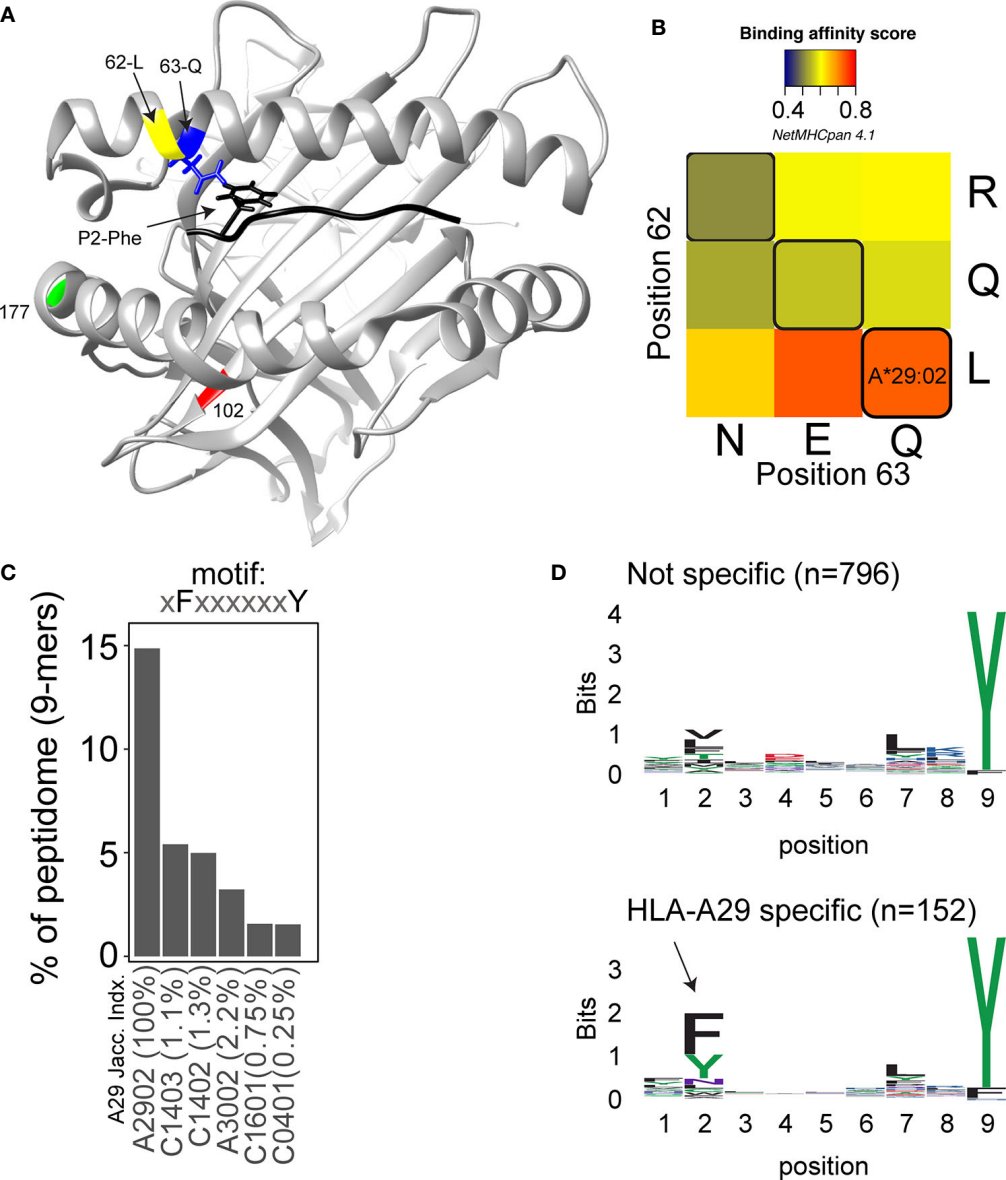
Structure and function of amino acid positions 62 and 63 in HLA-A29. **(A)** View into the peptide‐binding groove of a three‐dimensional ribbon model for HLA-A29 (Based on Protein Data Bank entry: 6J1W modelling using UCSF Chimera (42). The amino acids Leucine (L in yellow) at position 62 and Glutamine (Q in blue) at position 63 defining HLA-A29 are indicated. The binding peptide is shown in black with phenylalanine at position 2 (P2-Phe) interacting with position 63-Q (with energy-minimized positions of side chains). Polymorphic amino acid positions associated with the alleles *HLA-A29:01* (pos 102 in red) and *HLA-A29:10* (position 177 in green) are also shown. **(B)** The effect of amino acid substitutions for position 62 and 63 on predicted binding affinity for HLA-A29-presented peptides. The average binding scores of 9-mers (n = 948) detected by mass-spectrometry analysis of HLA-A29 reported by Venema et al. (43). Replacement of position 62 and 63 with the most commonly occurring amino acids at that position encoded by *HLA-A* alleles was done in netMHCpan 4.1 server (44). Naturally occurring motifs are indicated with black lines, other motifs (e.g., QN) do not occur in human HLA-A allotypes. **(C)** The percentage of 9-mer peptides with P2-Phe and P9-Tyr detected in immunopeptidomes of HLA class I alleles as reported by Sarkizova et al. (45). The top 5 (of 95 alleles tested) class I alleles other than HLA-A29 are shown. The jaccard similarity index for the HLA-A*29:02 peptidome (overlap in presented peptides) and each allele is indicated (in %). Peptidome data were derived from Sarkizova et al. (45). **(D)** Sequence logos of 9-mers (n = 948) from HLA-A29 [the same peptides as in **(C)**] stratified into non-specific for HLA-A29 [with binding score MSi > 0.6 for HLA-A*43:01 or HLA-A*68:130 according to the *HLAthena* server (45)] or specific for HLA-A29 (MSi < 0.6 for HLA-A*43:01 and HLA-A*68:130). The arrow indicates the aromatic P2 in the binding motif specific for HLA-A29.

**Figure 2:**
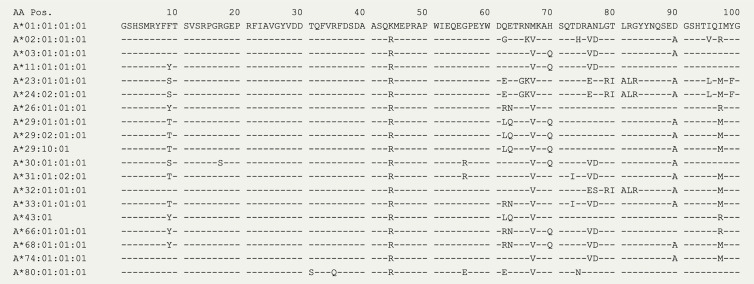
The amino acid sequence of HLA-A alleles. The first 100 amino acids for 19 HLA-A alleles from the IPD-IMGT/HLA Database (40). The amino acids at positions 62 and 63 distinguish HLA-A29 alleles, with the exception of the rare HLA-A43 (± 15,000 times lower allele frequency compared to *HLA-A29:02* in the European population).

In 2020, the *immuno polymorphism database* (40) contains >200 reported HLA-A29 alleles, but only the most common alleles—HLA-A*29:02, HLA-A*29:01, and HLA-A*29:10—have been reported in cases with birdshot (41). Structurally, HLA-A*29:01 (D102H) and HLA-A*29:10 (E177K) differ from HLA-A*29:02 at single amino acids positions in the external alpha 2 domain of HLA-A29 (**Figure 1A**), but these positions do not influence the expression, conformation, or interaction of the HLA-A complex with T cells (46–48). In other words, these alleles can be considered functionally similar. The most relevant amino acid positions in HLA-A29 for disease risk were statistically linked to amino acids at positions 62 and 63 in the protein sequence (33). As shown in **Figure 2**, the amino acids *Leucine* at position 62 (62-L) and *Glutamine* at position 63 (63-Q) distinguish HLA-A29 from other *HLA-A* alleles. This is of interest because computational modelling of HLA-A by changing amino acids at indicated positions (or amino acid substitution modelling) revealed that position 63 has the largest effect on the ability to bind antigenic peptides over all polymorphic positions in the peptide-binding “groove” of HLA-A *allotypes* (the term for “protein variants”) (49). Specific mutation of the positions 62-63 can completely abrogate HLA-peptide recognition by T cells (50). Most other *HLA-A* alleles encode the amino acids *asparagine* (N) or *glutamic acid* (E) at amino acid position 63 (**Figure 2**). Despite the degree of similarity of the chemical characteristics of the side chains of the amino acids, the effects of Q and N on the local structure of protein are different (51) and changing the chemically related glutamine (Q) to glutamic acid (E) at single amino acid position in the HLA-A molecule can modulate the interaction with CD8 of T cells (52). Indeed, amino acid substitution modelling of position 62 and 63 in HLA-A29 demonstrates that the strength of binding of peptides (i.e., the binding “affinity”) into the peptide-binding groove of HLA-A29 is decreased if the amino acids at these positions are changed to any of the other other naturally occurring combinations of amino acids at positions 62-63 in HLA-A (**Figure 1B**). Curiously, substituting position 62 is predicted to have a larger effect than substitutions on position 63. Also, the ‘theoretical’ motif 62-L 63-E (which does not occur in any known *HLA-A* alleles) provides a globally similar binding capacity for peptides compared to the 62-L 63-Q of HLA-A29. Phylogenetically related alleles of HLA-A29 (i.e., *HLA-Aw19* complex) (53) encode 62-Q 63-E and would require changing ‘only’ position 62 to achieve a globally similar functionality. However, as mentioned, the local structural effects of the chemically related E and Q can be quite distinct and functional analysis is required to better understand the hierarchy of impact of these positions on defining the HLA-A29 peptidome. Also, these amino acids do not completely account for the peptide specificity of HLA-A29. In fact, 62-L 63-Q is detected in some other alleles such as HLA-A*43:01, HLA-A*11:11, and HLA-A*68:130 [allele frequency of HLA-A*43:01 and HLA-A*11:11 in the European population >15,000 times less than HLA-A*29:02 (54), and HLA-A*68:130 is not well documented (40)]. However, these alleles differ from HLA-A29 alleles on various other key positions that influence peptide binding in the peptide-binding groove, including amino acid position 9 (55), 70, 76, 77, or positions 97 or 152, which influence the interaction with T cells (56, 57). Notwithstanding these exceptional alleles, the amino acid motif 62-L 63-Q near exclusively accounts for HLA-A29 in the European population (54). Amino acid residues 62 and 63 are positioned at the edge of the peptide-binding groove (**Figure 1A**) in a cavity that directly interacts with the side chain of the amino acid at position 2 (P2) of the displayed antigenic peptide (58). Therefore, the 62-63 motif may influence the flexibility to accommodate antigenic peptides with distinct P2 residues, a feature most likely relevant to autoantigen discovery for BU.

## The HLA-A29 Peptide Motif

The peptide binding motif—or the ‘conserved’ positional residue preference considering the amino acid sequences of all “presented” peptides—of HLA-A29 is relatively flexible on condition of a C-terminal (the last amino acid in a peptide) *Tyrosine* (Y) or less frequently a *Phenylalanine* (F) (45). Peptides with a C-terminal Y also make up a significant proportion of the peptides presented on other HLA-A alleles, including HLA-A*01:01, HLA-A*03:01, and HLA-A*30:02 (45) (but also HLA-A*43:01 and HLA-A*68:130). This includes peptides that are detected in the binding groove of more than one HLA-A allotype (demonstrated by mass-spectrometry studies of single-HLA-expressing cell lines), a phenomenon termed peptide ‘promiscuity’ (59). We and others have studied the complete set of antigenic peptides (termed the ‘immunopeptidome’) bound by HLA-A29 and used multidimensional scaling (a visual representation of the immunopeptidome where all peptides are positioned in a graph based on their relative similarity or difference in amino acid sequence) to cluster the peptides into subdominant binding motifs (or “submotifs”) (43, 45, 60). This approach facilitates the identification of clusters of antigenic peptides (submotifs) that are shared with other HLA class allotypes or that are unique to HLA-A29. These studies revealed patterns of submotif preferences easy to miss in conventional studies when considering the immunopeptidome as a whole. In short, HLA-A29 presents a palette of submotifs mostly defined by distinct amino acids at position 2 (P2) and 7 (P7) in the antigenic peptide sequence. As discussed, a fraction of peptides presented by HLA-A29 is also found in the binding groove of other HLA-A alleles and consequently some submotifs of HLA-A29 were also detected in immunopeptidome data of other HLA allotypes. This helped to narrow down a submotif that is specific to HLA-A29 (43), which is characterized by the amino acids F or Y at P2 in conjunction with the HLA-A29-characteristic C-terminal (PC) Y (F/Y-P2 + Y-PC motif). This motif makes up ~15% of the HLA-A29 immunopeptidome (**Figure 1C**). Peptides with this motif are substantially less frequently presented on other HLA allotypes and those that do are uncommon in the Western-European population and/or display very low similarity in the immunopeptidome composition with HLA-A29 (<3% of the peptides are shared, **Figure 1C**). Note that ‘just’ P2-F and P2-Y (so without PC-Y) is not uncommon in the immunopeptidomes of other HLA-A allotypes, such as HLA-A24 [see supplemental data of Sarkizova et al. (45)], but the amino acids that occupy the pocket accommodating P2 of binding peptides is completely different from HLA-A29. Although we were unable to find immunopeptidome studies of the HLA-A*43:01 and HLA-A*68:130 alleles, binding prediction shows that peptides with the motif F/Y-P2 + Y-PC are poorly presented by these alleles, most likely as a consequence of the differences in other key positions in the binding groove (**Figure 1D**), which further support that this motif is specific to HLA-A29.

## *The ERAP1*-*ERAP2* Haplotype Links BU to the Western-European Ancestry

**Figure 3:**
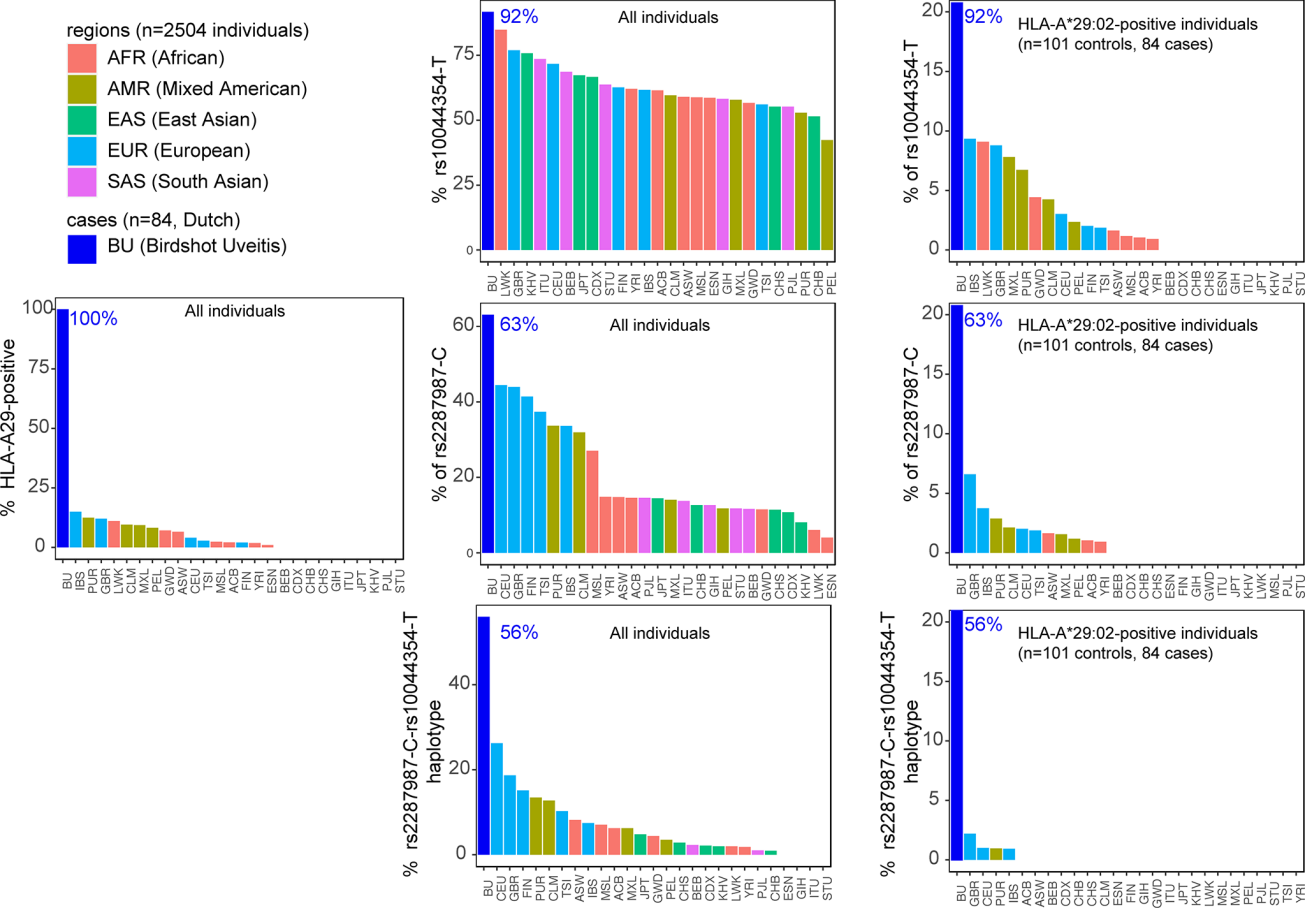
The combined risk factors HLA-A29 and the ERAP1-ERAP2 haplotype are restricted to populations of Western-European ancestry. The percentage of individuals that carry a copy of HLA-A*29:02, the C allele of the polymorphism rs2287987 (in *ERAP1*), and the T allele of rs10044354 (near *ERAP2*) in 84 BU patients and the 2504 individuals of 26 ethnic populations of the 1000 Genomes Project. Data from BU patients was derived from (61). The graphs in the middle are the data for all 2504 individuals of the 1000 Genomes. The graphs on the right are the genotype data limited to HLA-A*29:02-positive individuals. This data demonstrates that the combined risk haplotype of rs2287987-rs10044354 in HLA-A29 is rare or absent in populations of non-Western-European ancestry. HLA data was obtained from (39) and genotype data for rs10044354 and rs22878987 from the 1000 Genomes project (63). The regions and populations are indicated using the following abbreviations: CHB, Han Chinese in Beijing; JPT, Japanese in Tokyo; CHS, Southern Han Chinese; CDX, Chinese Dai in Xishuangbanna; KHV, Kinh in Ho Chi Minh City; CEU, Utah Residents (CEPH) with Northern and Western European Ancestry; TSI, Tuscans in Italy; FIN, Finnish in Finland; GBR, British in England and Scotland; IBS, Iberian Population in Spain; YRI, Yoruba in Ibadan; LWK, Luhya in Webuye; GWD, Gambian in Western Divisions in the Gambia; MSL, Mende in Sierra Leone; ESN, Esan in Nigeria; ASW, Americans of African Ancestry in SW USA; ACB, African Caribbeans in Barbados; MXL, Mexican Ancestry from Los Angeles USA; PUR, Puerto Ricans from Puerto Rico; CLM, Colombians from Medellin; PEL, Peruvians from Lima; GIH, Gujarati Indian from Houston; PJL, Punjabi from Lahore; BEB, Bengali from Bangladesh; STU, Sri Lankan Tamil from the UK; ITU, Indian Telugu from the UK.

**Figure 4:**
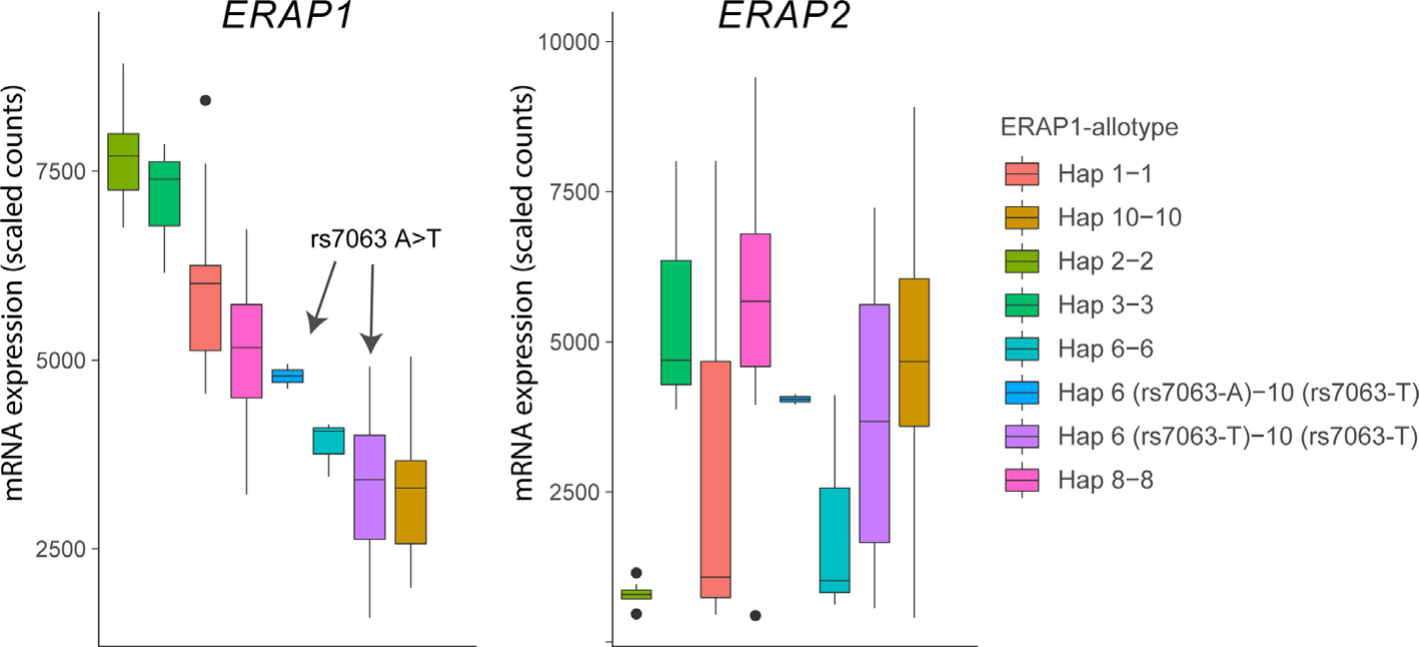
The splice associated variant rs7063 associated with haplotype 6 (97% of all *Hap6*) and 10 (99% of all Hap10) of *ERAP1* mediates low expression of these haplotypes in lymphoblastoid cells. Gene expression data for *ERAP1* and *ERAP2* from 85 homozygous or heterozygous cell lines with indicated haplotypes (and rs7063 allele) were obtained from available RNA-sequencing data of 358 lymphoblastoid cell lines from European ancestry of the GEUVADIS cohort (loaded using the *recount* R package) (62). Note that in cell lines that harbor the haplotype 10 containing the T allele of rs7063 genotype, the rs7063 genotype in Hap6 governs the expression of total *ERAP1* and that Hap6/Hap10 cell lines homozygous for the T allele of rs7063 show similar low expression compared to Hap10/Hap10 cell lines. The gene expression pattern for *ERAP2* (left plot) in the same samples does not mimic ERAP1 gene expression patterns, but reflect the non-random distribution of the ERAP2-protein coding haplotype across common *ERAP1* haplotypes, as we previously described (61). In particular haplotype 2 (Hap2; encoding *allotype 2*) is found infrequently in conjunction with ERAP2 haplotype A and, thus, shows low overall expression for ERAP2 in the *Hap2* homozygous cell lines in this example (n = 9).

Key to progress in understanding why merely a fraction of HLA-A29-positive individuals develop BU came from genetic studies, including work from our lab. We identified that beyond HLA-A29, genetic polymorphisms (or common variations in the DNA sequence among individuals) at chromosome *5q15* confer strong disease risk (34, 61). The signal on chromosome 5 covers the endoplasmic reticulum aminopeptidase (*ERAP*)-1 and *ERAP2* genes, and *LNPEP*, all enzymes involved in trimming the peptide fragments before they are bound by HLA class I (e.g., HLA-A29). Importantly, the combination of two polymorphisms functionally linked to *ERAP1* (rs2287987) and *ERAP2* (rs10044354), conferred a risk for BU that was significantly larger than the risk from either one the two polymorphisms individually (61). Analysis of patients and HLA-A29-positive controls showed that the combined polymorphisms linked to *ERAP1* and *ERAP2* also showed the largest disease risk (detected in 50% of 130 cases and 25% of 439 HLA-A29-positive controls). This indicates that the genetic changes affecting both ERAP1 and ERAP2 in tandem increase the risk in the HLA-A29-positive population (61). Indeed, if we look at publicly available data from the 1000 genomes project, the risk-variant linked to *ERAP2* (rs10044354-T) or the risk-variant linked to *ERAP1* (rs2287987-C) are also observed in HLA-A29-positive individuals of non-European ancestry (**Figure 4**). For example, most of the HLA-A29-positive cases in the Luhya in Webuye (LWT in **Figure 3**) of Kenya also carry the BU risk-variant near *ERAP2*, a population where no BU has been reported. In contrast, the combined risk polymorphisms are only observed in HLA-A29-positive individuals in Western-European populations in which BU is “endemic”, with the exception of Puerto Ricans (PUR in **Figure 4**). However, the majority (>60%) of Puerto Ricans is of European ancestry and the samples from this population were collected throughout the entire country (i.e., predominantly Caucasian) (64). Furthermore, BU is reported in Puerto Ricans (several Puerto Rican BU cases are reported in the *Retina Image Bank;* file numbers 6191 and 6178) (65). In contrast, the Tuscany population (TSI in **Figure 4**) includes samples collected from a small town near Florence in Italy. In this population the combination of HLA-A29 and the ERAP1-ERAP2 polymorphisms is rare. These data do not necessarily represent all individuals of a population of a country that is of mixed ancestry [e.g., BU is reported in Northern Italy (66)], but serves to explain why BU is very rare or non-existing in populations where the genetic combination associated with BU risk is exceptional.

This also implies that the ERAP1, ERAP2, and HLA-A29 collectively drive the pathogenesis of BU. We do like to emphasize that the number of individuals “burdened” with the “birdshot-genotype” still exceeds the estimated cases in each population (~1% of people from Western-European ancestry, of which about 1 in 500 develop BU as a rough estimate). Here, it is good to consider that the cause of HLA-A29-dependent BU is most likely heterogenous and in some patients may be mediated by genetic susceptibility imprinted in *ERAP* genes, while in others ERAPs may be dysregulated by alternative mechanisms. For example, ERAP1 is tightly regulated by TNF-alpha, a pro-inflammatory cytokine that is increased in concentration in eye fluids of BU patients and blocking TNF-alpha by anti-TNF therapy alleviates severe symptoms of BU (67–70). Polymorphisms linked to *ERAP1* and *ERAP2* genes are also associated with other HLA class I associated conditions that manifest with non-infectious uveitis, including HLA-B27-associated anterior uveitis and ankylosing spondylitis, or HLA-B51-associated Behcet’s disease (71–73). This supports the interdependence of ERAPs and HLA class I in the pathophysiology of non-infectious uveitis.

## ERAP1 and ERAP2 in the Antigen Presentation Pathway

The antigen presenting pathway for HLA(-A) starts with the degradation of cellular proteins (proteolysis) by the proteasome, a continuous and normal process to eliminate dysfunctional or mature proteins into shorter peptides (26, 27). A selection of peptides up to 20 amino acids long are transported into the endoplasmic reticulum (ER) by the *Transporter associated with antigen processing* (or TAP) and trimmed to generally 8-10 amino acid long peptides by ERAP1 and ERAP2 (26, 27, 74). ERAP1 and ERAP2 are specialized aminopeptidases that reside in ER (hence their name) where they each trim a proportion of the antigenic peptide pool before the peptides bind to HLA class I molecules for presentation at the cell surface (74). Structural studies have shown that the trimming process involves sequestering the entire peptide sequence inside the enzyme’s cavity after which the N-terminal (first amino acid in the peptide sequence) is trimmed off (75, 76), which indicates that the majority of peptides are trimmed before binding (77) to HLA class I (e.g. HLA-A29). Some HLA class II alleles lack protection of their peptide-binding groove when exposed to peptides in the endoplasmic reticulum and bind these peptides before their transportation to the cell surface (78). It’s not unlikely that ERAPs may influence the peptide cargo presented by a selective group of these ‘unprotected’ HLA class II alleles, but this is unexplored. Also, ‘free’ ERAP1 and ERAP2 is found in body fluids and blood (79), and can be secreted by stimulated immune cells (80). Notwithstanding these other functions, the strong genetic association with HLA-A29-associated BU implicates ERAP-dependent antigen presentation by HLA-A29 as a key disease pathway in BU. To better understand how ERAP1 and ERAP2 modulate HLA-A29, let’s first detail the genetic associations mapped to these genes.

## ERAP1 and Birdshot

The *ERAP1* gene encodes various distinct ERAP1 haplotypes (a group of polymorphisms inherited together) that encode functionally different protein variants (which are termed “allotypes”) with markedly different capacity to cut antigen peptides in terms of speed and specificity (61, 81). Genetics studies showed that the C allele of the polymorphism rs2287987 was more frequently seen in patients with BU (61). The rs2287987-C is found almost exclusively in the haplotype (named *Hap10*) that encodes *Allotype 10* (61). Detailed description of ERAP1 polymorphisms and their effects on functions of ERAP1 can be found elsewhere (74, 82). Briefly, the minor C allele of rs2287987 located in exon 6 of ERAP1 results in the change of Methionine to Valine at amino acid position 349 in ERAP1. This amino acid change occurs in the active site of ERAP1 next to the hallmark zinc-binding motif of the family of M1 zinc metallopeptidases (H-E-X-X-H-(X)18-E motif) and affects the enzymatic activity of the enzyme. However, the rs2287987 bearing allotype 10 contains additional polymorphisms that indirectly affect the specificity or activity by changing the structural conformation of the enzyme (83, 84). Note that rs2287987-C is also common in African populations (**Figure 3**) but it often resides in ERAP1 haplotypes different from Hap10. Allotype 10 is characterized by enzymatic activity that is magnitudes lower compared to other characterized allotypes of ERAP1, but also shows relatively low expression (61). The latter feature is caused by moderate LD between the C allele of rs2287987 and a splice interfering variant [T allele of rs7036 (85)] located upstream in an untranslated region of *ERAP1* [rs7063 is present in >90% of *Hap10* and typically <5–10% of other common haplotypes of *ERAP1* (61)]. Although low expression and low enzymatic activity of *Hap10* is often mentioned in the same breath in discussions of its pathogenic contribution to disease, rs2287987 is associated with BU independently from rs7063, thus, the BU risk linked to rs2287987 represents more likely the diminished enzymatic activity of ERAP1 (61). In fact, rs7063-T is also found in >90% of another common haplotype of ERAP1 named Hap6 (61).

Because the vast majority of Hap6 shares the T allele of rs7063 with Hap10 (61), gene expression data from the 1000 Genomes project shows that consequently the expression of the two haplotypes is comparably low (**Figure 4**). Although the ERAP1 expression levels have been shown to influence the immunopeptidome of HLA-A29 (86), the low expressed Hap6 is not associated with BU (61) and makes it more plausible that the highly distinct enzymatic features of Hap10 contribute to the susceptibility to BU. This is further supported by the fact that the highly active ERAP1 allotype encoded by *Hap2*, the functional “antagonist” of Hap10, is protective against BU (61). Because of these differences in trimming capacity, the lack of destruction of a uveitogenic epitope is a plausible mechanism for disease.

## ERAP2 and Birdshot

Of the two common haplotypes (A and B) of *ERAP2* detected in the population*, haplotype A* (HapA) encodes the canonical full-length ERAP2 protein (87), but is consists of many polymorphisms that are located also far outside *ERAP2* and deep into the *LNPEP* gene. The polymorphisms of *HapA* outside *ERAP2* are located in the intragenic regions of the *LNPEP* gene and are not encoded into the *LNPEP* gene products. Haplotype B (HapB) contains a polymorphism (the G allele of rs2248734) that changes a splice region and facilitates intronic read-through until a stop codon, which under steady state conditions targets the transcript for destruction and results in barely detectable levels of the full-length ERAP2 protein (87). However, HapB has been shown to produce a truncated ERAP2 protein in response to infection by various microbial pathogens, interferon alpha or bacterial lipopolysaccharides (88–90). The strongest association at *5q15* is linked to the polymorphisms in HapA (tagged by rs10044354 in an intragenic location of *LNPEP*). We showed that this signal did not influence the splicing or expression of the *LNPEP* gene, but showed that high ERAP2 expression controlled by this genetic signal embedded in HapA is a risk factor for BU (34, 61). Here we note that the polymorphism rs10044354 (and variants in LD) is independently associated with BU from the polymorphism rs2248374 that governs splicing of *ERAP2* into its main haplotypes (61). Data from the *The Genotype-Tissue Expression (GTEx) project* supports that rs10044354 is strongly associated with the expression of *ERAP2* and mildly impacts the expression of *LNPEP* across various tissues (91). However, the effect sizes of rs10044354 on the expression of other nearby genes *ERAP1* and *CAST* are in the same range as for *LNPEP* and of unknown biological significance. In summary, high ERAP2 expression is a significant risk factor for BU in HLA-A29-positive individuals. The generation of *uveitogenic* peptides by ERAP2, which hypoactive ERAP1 fails to destroy, is a plausible disease mechanism for BU.

## ERAP1 and ERAP2 Shape the HLA-A29 Immunopeptidome

Both ERAP1 and ERAP2 have been shown to affect the HLA-A29 peptidome of cell model systems (86, 92), which has been reviewed in detail elsewhere (74). In short, by global assessment of the immunopeptidome, active ERAP1 allotypes (e.g. *Hap2*) decrease the length of peptides of 10 amino acids or longer (10-mers), with a net increase of 9-mers (86). In the presence of active ERAP1 allotypes, the number of peptides with phenylalanine (F) and tyrosine (Y) at the first two amino acids of the peptide sequence (the N-terminal position 1 [P1] or 2 [P2]) of the binding peptides was slightly increased (86). ERAP2 shapes the HLA-A29 immunopeptidome predominantly by destruction of peptides with a P1 amino acid that are preferred substrates for ERAP2, predominantly Lysine (K), Arginine (R), and Alanine (A) (93). Large aromatic amino acids F and Y are poor substrates for ERAP2. Because ERAP2 destroys competing peptides that harbor optimal residues (K, R, or A at P1) for ERAP2, peptides that contain F or Y at P1 consequently make up a relatively larger proportion of the available antigen peptides to bind HLA-A29 and become “over-represented” in the presence of ERAP2 (43, 92). Other studies from the López de Castro group also demonstrated these ERAP2 effects on the immunopeptidome of risk HLA allotypes of other types of uveitis, such as HLA-B27 (94). In a recent study, we demonstrated that the effect of ERAP2 on P1 is actually a common feature of ERAP2 observable in the immunopeptidomes of all HLA class I alleles expressed by the cell (43). Intriguingly, Abelin and coworkers showed that peptides presented by HLA class I allotypes show a depletion for K, R and A residues at P1, which we believe can be attributed to the fact that their studied cell line expresses ERAP2 (60). So perhaps ERAP2 is evolutionary designed to destroy epitopes with these characteristics as a means to lower the immunogenicity of the presented immunopeptidome. This hypothesis comes from the observation that cancer patients with high ERAP2 expression showed worse overall survival after checkpoint inhibitor therapy (allowing T cells to kill cancer cells), relative to those with low ERAP2 expression (95). In other words, when T cells are “licensed” to attack tumors unrestrictedly, immune responses are more dependent on the level of antigen presentation to T cells. Here, ERAP2 may influence the HLA class I immunopeptidome so it provides less T cell epitopes to destroy tumors (and perhaps normal tissue, but this was not evaluated). This may in part be explained by the fact that the side chains of the amino acids at P1 influence the spatial configuration of amino acid position 167 in HLA-A, which has been shown to tune the peptide recognition by T cells and affect the peptide binding repertoire (49, 96). Here, the amino acids K and R induce a similar configuration of position 167 that is distinct from the configuration the alpha domain adapts at this position if P1 contains a F or Y (96) which functionally parallels the preference for trimming these amino acids by ERAP2. Beyond the universal effects on P1, peptides that are destroyed by ERAP2 may also share additional characteristics. For example, ERAP2 also showed preference for amino acids at position 3 and 7 in the antigenic peptide (43), which matches the pockets of ERAP2 that would interact with the sidechain of these residues (75). Similar to the effects of P1, as mentioned, the destruction of HLA-A29 epitopes by ERAP2 most likely represents a canonical function of ERAP2.

## The ERAP1-ERAP2 Risk Haplotype Exhibits HLA-A29-Specific Effects

In contrast to the shared effects of ERAP2 across HLA class I immunopeptidomes, in patient derived cell lines homozygous for the risk ERAP1 allotype *Hap10*, ERAP2 facilitated the increased presentation of peptides with F or Y at P2 specifically for HLA-A29 (43, 92). Because this is the same submotif that distinguishes HLA-A29 from other HLA class I alleles, this observation provides a possible explanation for the association of these genes with BU. These submotif-specific effects of ERAP2 were also detected in different source data regarding immunopeptidomics of HLA-A29 (43). This indicates these effects of ERAP2 on HLA-A29 are generalizable, but may also help narrow down the putative disease modifying effects of the antigen presentation pathway. Recall that active ERAP1 allotypes (other than *Hap10* and in ERAP2-deficient cells) also showed a moderate increase of F at P2 in the HLA-A29 immunopeptidome (86). However, submotif analysis of this data revealed that ERAP1 did not bias the immunopeptidome in favor of the HLA-A29-specific submotif like ERAP (43). This may be because the pocket in which the side chain of P2 binds in ERAP2 has limited space which doesn’t allow bulky aromatic residues (75), while the analogous pocket in which P2 would bind in ERAP1 is more open and could accommodate bulky residues to some degree (i.e., F or Y) (76). Furthermore, considering individual peptides, there is low correlation between the effects of ERAP1 and ERAP2 on HLA-A29 (43), which indicates context specificity and possible non-redundant pathogenic contributions to antigen presentation by HLA-A29 that increase the risk for BU. Such independent pathogenic contributions by ERAP1 and ERAP2 are supported by the genetic studies (61) of BU as discussed.

## HLA-A29 and Autoantigen Presentation

Given that HLA-A29 is prerequisite for the development of BU, we hypothesize that disease mechanisms associated with antigen presentation are most likely driven by a limited set of epitopes (or single peptide) because of promiscuity of peptides across HLA class I (59). Based on the submotif that is specific to HLA-A29 and supported by ERAP2, we hypothesize that ‘uveitogenic’ HLA-A29-restricted peptides may more likely harbor a F or Y at P2 (and a Y at PC). The importance of P2 in HLA-A29-restricted peptides is in line with the fact that the amino acids at position 62-63—which define HLA-A29—directly interact with P2 of the binding peptide (**Figure 1**). The hypoactive ERAP1 allotype strongly linked to BU predominantly may prevent the destruction of 9-mers or longer peptides (43, 73, 92). There are examples of HLA-A29-presented 10-mers that cause strong T cell-mediated responses in humans, such as AELLNIPFLY encoded by *UGT2B17* (97, 98).

Curiously, the HLA-A*29 alleles are among the lowest expressed *HLA-A* alleles (99). However, the amount of peptides presented by HLA class I only weakly correlates with HLA levels at the cell surface, plus immunopeptidome studies support that HLA-A29 is potent in presentation of peptides at the cell surface (43, 45, 92). Of interest, HLA class I is generally low expressed in the retina (100), while HLA class I expression is relatively high around endothelial cells of large vessels of the choroid (101), the presumed epicenter of inflammation in BU (12). Choroid melanocytes are densely located around these endothelial cells and have been proposed as an autoantigen-source in BU etiology (1). Perhaps BU is driven by HLA-A29-presented ERAP-dependent melanocyte-derived peptides (1, 61). This also fits the “*autoimmune surveillance of hypersecreting mutants*” (ASHM) theory, which predicts that autoantigens involved in organ-specific autoimmunity (the eye) should be linked to secreting cells such as melanocytes (102), where autoimmunity is considered a natural tradeoff to prevent lethal disease mediated by hypersecreting mutants. Besides their more commonly known role in pigment production, choroid melanocytes have also been shown to contribute to the maintenance of the normal vasculature structure of the choroid (103). Of interest, melanocytes can produce powerful angiogenic factors upon suppression of tyrosinase activity, the key enzyme in pigment production (104). It is conceivable that proteins highly expressed in choroidal melanocytes are closely monitored by surveilling self-reactive T cells (following the ASHM theory), because of the potential devastating effects of hypersecreting mutants, at the cost of autoimmunity. The autoimmune conditions VKH, *vitiligo*, and *psoriasis* are a proof of principle that melanocytes harbour autoantigens that are targets for autoreactive T cells (105–108). Gene expression or proteomic studies comparing cutaneous and choroid melanocytes are warranted to understand their potential differences to better understand the restriction of BU inflammation to the eye. Of interest, the ERAP2-promoted HLA-A29-specific peptide motif (P2-F + PC-Y) is observed in the amino acid sequence of key proteins of melanogenesis that are expressed in the eye (43). These include a number of putative candidate peptides from melanocyte proteins, such as CFVALFVRY (SLC45A2), CFPLLRLLY (OCA2), or SFSKLLLPY (PLXNC1) (43). Of course, functional experiments are required to validate if any of these ‘potential’ peptides are actually presented by HLA-A29. As mentioned, the circumstantial association with melanoma (i.e., a ‘hyper’ anti-melanoma response) has sparked interest for this theory, but lacks evidence for any causal relation (109). Remarkably, although HLA-A29 can effectively present melanoma epitopes (1), HLA-A29 is associated with worse survival compared to HLA-A29-negative melanoma patients (110). This could indicate that perhaps similar to the effects of ERAP2, in general, HLA-A29 and ERAP2 may ‘lower’ the immunogenic peptide cargo presented to T cells, but only increase the expression of a very limited (perhaps single) antigen under specific conditions that cause BU. Alternatively, the loss of choroidal melanocytes may be collateral damage from dysfunction in the choroidal endothelium. More specifically, the disruption of a *Hedgehog*-signaling axis from choroidal endothelial cells to choroidal stromal cells (i.e., perivascular mesenchymal stem cell-like cells that suppress T cell function) resulted in the loss of choroid melanocytes and illustrates a key role for the choroidal endothelium for the maintenance of choroidal immune homeostasis (111).

The retinal S-antigen has long been considered as a major autoantigen for BU, because S-antigen causes a birdshot-like phenotype in primate models (112, 113) and T cells from patients proliferate after stimulation with S-antigen (24, 114). However, S-antigen immune reactivity is widespread among clinically distinct phenotypes of uveitis and linked to T helper cell responses (linked to HLA class II), which suggests it plays a role in BU independent of HLA-A29, perhaps at later stages of the disease. This is in line with the retinal lesions observed in BU patients and suggests the retinal S-antigen may have a role at the clinical stage. However, also other retinal proteins contain peptides that may be presented by HLA-A29 (109). Previous *in vitro* studies determined that peptides derived from the S-antigen can bind to HLA-A29 (115), but further research using immunopeptidomics of cells expressing S-antigen are required to define the HLA-A29 presented epitopes of S-antigen. We recently identified that a naturally HLA-A29-presented peptide of S-antigen is VTLTCAFRY and currently assess if this peptide is also recognized by T cells of patients (43).

## The Microbiome, Th17 Cells, and HLA-A29

The *commensal microbiome* is a fast universe of diverse and mostly uncharacterized microbial species which inhabit tissues such as the skin and gastrointestinal tract where they collectively influence the functions of the immune system (116). For example, CD8+ T cells are primed by microbial derived metabolites and MHC-I presented microbial derived peptides to cross-react with cancer antigens as a means to facilitate anti-tumor immunity (117, 118). Gut microbiome dysbiosis is observed in patients with inflammatory conditions and considered to cause disturbance of systemic immune homeostasis in uveitis (119). In animal models, gut commensals have been shown to directly activate T helper 17 (Th17) cells to trigger uveitis (120).

This is of interest, because BU patients show increased numbers of blood Th17 cells and elevated levels of Th17-cytokines (68, 121–123). Of interest, Th17 cells induced by infection such as the fungal commensal *C*. *Albicans* may persist and aggravate autoimmune disease of the kidney (124). Protective anti-*C. albicans* responses by Th17 cells have also paradoxically been shown to result in inflammatory lung disease or inflammatory bowel disease in some individuals (125). Although *C. albicans* infection can affect the choroid and retina in a small percentage of patients (126), this shows a very different clinical phenotype. Regardless, the Th17-signature in BU could be related to changes in the microbiome. Studies of the microbiome of patients with BU are not yet conducted, the first study of HLA-A29-positive individuals as a whole show show a distinct intestinal microbiome composition (127) as demonstrated for HLA-B27-positive or HLA-DRB1-positive controls (128). In fact, microbiome similarity is observed in individuals who shared HLA alleles (129), which suggests that HLA influences the composition of the gut microbiome in part as a canonical feature of the immune homeostasis. The interaction of the microbiome in antigen-presentation *via* HLA-A29 in the disease mechanisms of BU requires further investigation, ideally by integrating the novel insights from studies of ERAP1 and ERAP2. Of interest, HLA class I bound by *Killer immunoglobulin-Like Recepto*rs (KIRs) on T cells promotes the expansion of Th17 cells in patients with HLA-B27 pathologies (130). Furthermore, specific modulation of ERAP1 has been shown to influence Th17 expansion (131). Therefore, it would be interesting to determine if similar biological mechanisms are linking HLA-A29 to Th17 responses in BU.

## KIR Receptors and Birdshot

BU may be driven by additional inflammatory genes since its genetic profile displayed shared genetic contributions with other inflammatory conditions, including *systemic lupus erythema* and *Neuromyelitis optica*, that both involve the eye (132). Among these may also be additional factors of the antigen presentation pathway, including the autophagy gene *TECPR2* previously reported (34) or *Killer immunoglobulin-Like Recepto*r (KIR) (KIRs) genes (133). KIR genes have been associated with BU, however, the allele frequencies of controls used in a study of BU patients may not be representative for European populations and stringent correction for multiple testing is required to avoid false-positives, which may influence the outcomes of *KIR* associations in BU (133, 134). Regardless, KIRs are important receptors for T cells, but also *Natural Killer* (NK) *cells*, an understudied immune cell in the context of non-infectious uveitis that is decreased in the circulation of BU patients (135). Curiously, immunosuppression therapy restores the number of NK cells in patients with uveitis (136). The role of KIRs in BU also merits further functional investigation because NK cells have been shown to get activated by HLA class I by altering the presented peptide (137). It will be interesting to explore the role of the *ERAP1-ERAP2* haplotype in peptide presentation by HLA-A29 and NK cell responses in patients. However, in HLA-B27-associated ankylosing spondylitis, the strong genetic interaction of *ERAP1* with HLA-B*27 was independent from genetic associations with *KIR* genes, suggesting that the disease mechanisms of ERAP and HLA class I may be mostly distinct from interaction of KIRs with HLA class I (138), and perhaps represent complementary mechanisms such as shown for free heavy chain expression by HLA-B27 and KIR interaction (131), while ERAP may mediated antigen-specific T cell responses. Indeed, T cell receptor (TCR) analysis of CD8+ T cells in patients with AS suggest differential antigen exposure (139) and similar studies of TCR repertoires of BU are currently underway.

## Concluding Remarks

In this review, we discussed how key features of the antigen presentation pathway predispose to eye-specific autoimmunity in BU. The prerequisite for HLA-A29 and the enrichment for functional polymorphisms that affect the function of antigen processing enzymes ERAP1 and ERAP2 point toward a key contribution for the antigen presentation pathway in the etiology of BU. Using functional studies, we are beginning to understand how ERAPs shape the immunopeptidome of HLA-A29 and a growing body of evidence is closing in on their disease modifying effects. This will help to better predict the outcome of pharmacological interference of ERAPs activity using newly available small molecule inhibitors (140) that may soon be applied as a high precision medicine to halt autoimmunity and restore eye health in patients, while leaving immunity toward pathogens and cancerous tissues intact.

## Author Contributions

All authors contributed to the article and approved the submitted version.

## Funding

JK is supported by a VENI award from the Netherlands Organization for Scientific Research (N.W.O. project number 016.186.006). WV is supported by UitZicht (project number 2018-1) and Stichting Lijf en Leven (project number 63). The funders had no role in the design, execution, interpretation, or writing of the study.

## Conflict of Interest

The authors declare that the research was conducted in the absence of any commercial or financial relationships that could be construed as a potential conflict of interest.
